# Disadvantageous Deck Selection in the Iowa Gambling Task: The Effect of Cognitive Load

**DOI:** 10.5964/ejop.v11i2.931

**Published:** 2015-05-29

**Authors:** Melissa J. Hawthorne, Benton H. Pierce

**Affiliations:** a Department of Psychology, Counseling, and Special Education, Texas A&M University-Commerce, Commerce, TX, USA; Aalborg University, Aalborg, Denmark

**Keywords:** Iowa Gambling Task, attention, frequency awareness, divided attention, decision making

## Abstract

Research has shown that cognitive load affects overall Iowa Gambling Task (IGT) performance, but it is unknown whether such load impacts the selection of the individual decks that correspond to gains or losses. Here, participants performed the IGT either in a full attention condition or while engaged in a number monitoring task to divide attention. Results showed that the full attention group was more aware of the magnitude of gains or losses for each draw (i.e., payoff awareness) than was the divided attention group. However, the divided attention group was more sensitive to the frequency of the losses (i.e., frequency awareness), as evidenced by their increased preference for Deck B, which is the large but infrequent loss deck. An analysis across blocks showed that the number monitoring group was consistently more aware of loss frequency, whereas the full attention group shifted between awareness of loss frequency and awareness of payoff amount. Furthermore, the full attention group was better able to weigh loss frequency and payoff amount when making deck selections. These findings support the notion that diminished cognitive resources may result in greater selection of Deck B, otherwise known as the prominent Deck B phenomenon.

The role of emotional processes in decision making has become an increasingly popular research topic in recent years. One of the most widely used instruments in the study of affect and decision making is the Iowa Gambling Task (IGT; Bechara, 2007; Bechara, Damasio, Damasio, & Anderson, 1994; Smith, Xiao, & Bechara, 2012). This task mimics real-life decision making situations that can be navigated successfully by healthy adults. However, patients with damage to the ventromedial prefrontal cortex (e.g., Bechara, Damasio, Damasio, & Lee, 1999; Bechara, Damasio, Tranel, & Damasio, 1997, 2005), the amygdala, (Bechara et al., 1999), the right parietal cortex (Tranel, Bechara, & Damasio, 2000), and the anterior and posterior orbitofrontal cortex (Bechara, 2007) show similar patterns of decision-making impairment on the task.

In the Iowa Gambling Task, participants are told that they have been loaned $2000 and are instructed to maximize their gains and minimize their losses. Participants are presented with four decks, each of which is either a good deck (i.e., advantageous) or a bad deck (i.e., disadvantageous). Within each deck, every card provides a monetary gain and a potential loss. The two bad decks (Decks A and B) give high immediate and constant rewards. However, these decks also have substantial losses, resulting in a net loss if participants draw frequently from those decks. The two good decks (Decks C and D) provide smaller consistent rewards, but the penalties are smaller than the rewards, resulting in an overall long-term gain (See Table 1 for deck characteristics). In addition, the decks differ in their payoff schemes. Two decks (Decks A and C) have frequent losses, whereas Decks B and D have infrequent losses. With each draw of the card, participants are presented with the amount gained, the amount lost, and a running total of their overall net gain or loss. Although the typical number of draws is 100, participants are not told this in advance. The purpose of the task is to determine if participants, through a trial-and-error process, learn to prefer the good decks.

**Table 1:** Gains and Losses on the Iowa Gambling Task

	Deck A	Deck B	Deck C	Deck D
Gains	$100	$100	$50	$50
Losses	$150-$350	$1250	$50	$250
Frequency of Gains/Losses (10 trials)	5:5	9:1	5:5	9:1

Although there has been a substantial body of research showing that healthy participants gradually shift their deck choices from Decks A and B to Decks C and D, these studies have used the traditional scoring method (Steingroever, Wetzels, Horstmann, Neumann, & Wagenmakers, 2013). This method calculates participants’ overall proportion of selections from the good decks, or alternatively, the difference in the overall proportion of choices between the good and bad decks. However, when IGT performance has been examined on a deck-by-deck basis, a different pattern emerges. In a meta-analysis of IGT studies, Dunn, Dalgliesh, and Lawrence (2006) observed that in some studies, both normal controls and clinical participants favored Decks B and D over Decks C and D. Similarly, Toplak, Jain, and Tannock (2005) showed that participants chose Deck B more often than the other decks. Indeed, several studies have demonstrated that rather than developing a preference for the advantageous decks, participants tended to develop a preference for one advantageous deck (Deck D) and one disadvantageous deck (Deck B), with participants often selecting more cards from Deck B than from the other decks (Toplak et al., 2005; Wilder, Weinberger, & Goldberg, 1998). This pattern of preferring Deck B over other more advantageous decks has been labeled the “prominent Deck B phenomenon” (Chiu, Lin, Huang, Lin, Lee, & Hsieh, 2008; Lin, Chiu, Lee, & Hsieh, 2007). Deck B is unique, in that it is the only deck that gives high immediate and constant rewards, but the losses (which are infrequent) heavily outweigh these gains, making it a bad deck in the long run. Although Deck A is also considered to be a bad deck in that over time the losses outweigh the gains, the individual losses are smaller (typically $150-$350) and more frequent (see Table 1).

Several studies have examined various factors that could impact IGT performance. Mood has been shown to affect performance, with a positive mood state leading to better performance compared to a negative mood state (e.g., Bagneux, Font, & Bollon, 2013; de Vries, Holland, & Witteman, 2008; Miu, Heilman, & Houser, 2008). Other research has explored the effect of instructional modifications, although the results have been mixed. For example, Balodis, MacDonald, and Olmstead (2006) found that when participants were provided a shortened version of the task instructions, the expected learning pattern failed to develop. However, Hawthorne, Weatherford, and Tochkov (2011) provided participants with instructions that varied in length and detail and found the traditional shift to advantageous decks in all three conditions. Finally, factors such as time limitations (Cella, Dymond, Cooper, & Turnbull, 2007; DeDonno & Demaree, 2008), have been shown to lower performance.

In order to more fully understand the cognitive processes underlying this preference for Deck B, Huizenga, Crone, and Jansen (2007) used a multilevel approach in examining developmental changes in IGT strategy. These authors used a developmentally appropriate version of the IGT (i.e., The Hungry Donkey Task) with school-aged children (ages 6-9), older children (ages 10-12), adolescents (ages 13-15) and university students (ages 18-25) to examine how performance strategies changed based on developmental maturation. These authors demonstrated that age-related improvement on the IGT can be explained by a shift from a unidimensional to a multidimensional proportional reasoning strategy. According to this perspective, IGT performance is influenced by three dimensions: (a) the amount of net gain, (b) the frequency of losses, and (c) the amount of probabilistic loss. Huizenga et al. hypothesized that the task is too complex for participants to analyze all three dimensions and so they rely on strategies based on a single dimension. For children, the relevant dimension tends to be loss frequency. With developmental gain comes the ability to consider more than one aspect of the IGT, resulting in a shift to multidimensional reasoning that includes loss frequency and the amount of probabilistic gain.

This shift in reasoning is evident in participants’ changing deck preferences on the IGT. For example, Huizenga et al. (2007) found that a focus on loss frequency was the default strategy for all age groups. However, a subgroup of participants, primarily older adolescents and adults, employed a more sophisticated approach, in which they focused first on loss frequency, but then also considered the amount of probabilistic loss. Huizenga et al. found that participants who relied solely on loss frequency favored Decks B and D, whereas participants who considered probabilistic loss as an additional factor tended to develop a preference for Deck D over Deck B as the task progressed. The authors also found that participants who used probabilistic loss as a factor had higher levels of inductive reasoning ability as measured by the Raven Standard Progressive Matrices test (Raven, Court, & Raven, 1985). Huizenga et al. further hypothesized that participants who focused only on loss frequency likely also had more limited processing capacity, and therefore were not able to consider other relevant factors.

Cassotti, Houdé, and Moutier (2011) found similar results with children and adolescents, who showed a strong preference for disadvantageous decks, but still chose Deck B more than Deck A. Although children, adolescents, and adults started out with similar patterns of deck selection, by the final block the patterns had changed. Children and adolescents drew more from Deck B, whereas adults selected more cards from Decks C and D. Furthermore, Beitz, Salthouse, and Davis (2014) found that children, adolescents, and older adults preferred decks with low loss frequency as evidenced by a greater number of draws from Deck B. Young and middle aged adults, however, developed a preference for decks with higher net outcomes (Decks C and D). These results are consistent with the recurring theme that certain populations are more susceptible to the prominent Deck B phenomenon. In general, populations whose cognitive abilities are not fully developed (e.g., children and adolescents) and those whose cognitive abilities have begun to decline (e.g., older adults) are more likely to favor Deck B (e.g., Beitz et al., 2014; Crone, Bunge, Latenstein, & van der Molen, 2005; Crone & van der Molen, 2004; Huizenga et al., 2007). Additionally, populations who suffer from compromised cognitive resources such as people with schizophrenia (Shurman, Horan, & Ntuechterlein, 2005) and those with ADHD (Toplak, Jain, & Tannock, 2005) also tend to favor Deck B over more advantageous decks.

Furthermore, research has shown that placing a load on executive functions may disrupt learning on the IGT. For example, Hinson, Jameson, and Whitney (2002) used both a digit load task and a random number generation task to demonstrate that working memory load interfered with IGT performance. In a later study, Jameson, Hinson, and Whitney (2004) found that IGT performance was compromised by having participants engage in a working memory task, but not by engaging in a verbal buffering task. The authors hypothesized that the verbal buffering task may not have sufficiently interfered with participants’ attentional resources to affect IGT performance. Similarly, Pecchinenda, Dretsch, and Chapman (2006), using a modified version of the IGT, found that low working memory load did not disrupt IGT performance, whereas high working memory load did impair performance. Overall, these findings suggest that working memory does play a role in IGT performance and that impaired working memory capacity can cause poor performance on the task (but see Manes et al., 2002). Other research that has attempted to identify cognitive resources necessary for optimal IGT performance has yielded mixed results (Beitz, Salthouse, & Davis, 2014; Toplak et al., 2010).

To provide further support for the notion that diminished cognitive resources, particularly attentional resources, may underlie the prominent Deck B phenomenon, the present study examined the effects of placing a cognitive load on IGT performance in healthy younger adults. Although we drew from Huizenga et al. (2007) in our discussion of strategies involved with the IGT, our focus was somewhat different. Rather than attempting to establish the impact of cognitive load on strategy use, we examined how such impairment affects awareness of certain aspects of the task and the resulting behavioral results. Participants completed the IGT under either a full attention condition or a number monitoring condition. Because there is a marked preference for Deck B among populations who have diminished cognitive resources, we expected that by placing healthy young adults in a divided attention condition, we would be able to produce a prominent Deck B effect.

## Method

### Participants

A total of 60 undergraduate students from Texas A&M University-Commerce participated in the study and were randomly assigned to one of two groups. Participants consisted of 38 females and 22 males. The mean age was 21.87 with a range from age 18 to age 29.

### Materials

#### Iowa Gambling Task

A computerized version of the IGT was used in this study. The computer screen displayed four rectangles with a number 1-4 in each rectangle. Participants were instructed to select a card by clicking on the appropriate rectangle. Below the rectangles, the card choice, reward, penalty and net gain were reported in green text. The participant’s total score was reported in black font. A green score bar at the bottom of the screen displayed the gains and losses. The reward structure and instructions were identical to those established by Bechara et al. (1994: see Table 1 for item characteristics). The task was self-paced.

#### Number monitoring task

Number monitoring tasks are often used in divided attention paradigms because they have low error rates but high processing demands that tend to disrupt encoding of information (Fernandes & Moscovitch, 2000; Park, Smith, Dudley, & Lafronza, 1989). In this task, participants heard numbers through a set of head phones and were asked to click a hand-held counter whenever they heard a number containing the number 3 (e.g., 13, 23, 33). The numbers were all spoken in a female voice and presented at 4 second intervals using Windows Media Player.

### Procedure

Participants were tested individually in a quiet room during a 45-minute time period. Before beginning the session, each participant completed an informed consent and a demographic form. The single-task group completed the IGT without engaging in a simultaneous secondary task. The number monitoring group received instructions for the number monitoring task and then completed the IGT and the secondary task simultaneously.

We calculated payoff (net outcome) and sensitivity to loss frequency using the Stocco et al. (2009) scoring method. That is, we determined payoff by subtracting the number of draws from bad decks from the number of draws from good decks (i.e., (Deck C + Deck D) - (Deck A + Deck B). We then calculated frequency sensitivity by subtracting the number of draws from decks with a low frequency of loss from decks with a high frequency of loss (i.e., (Deck B + Deck D) - (Deck A + Deck C).

## Results

**Figure 1:**
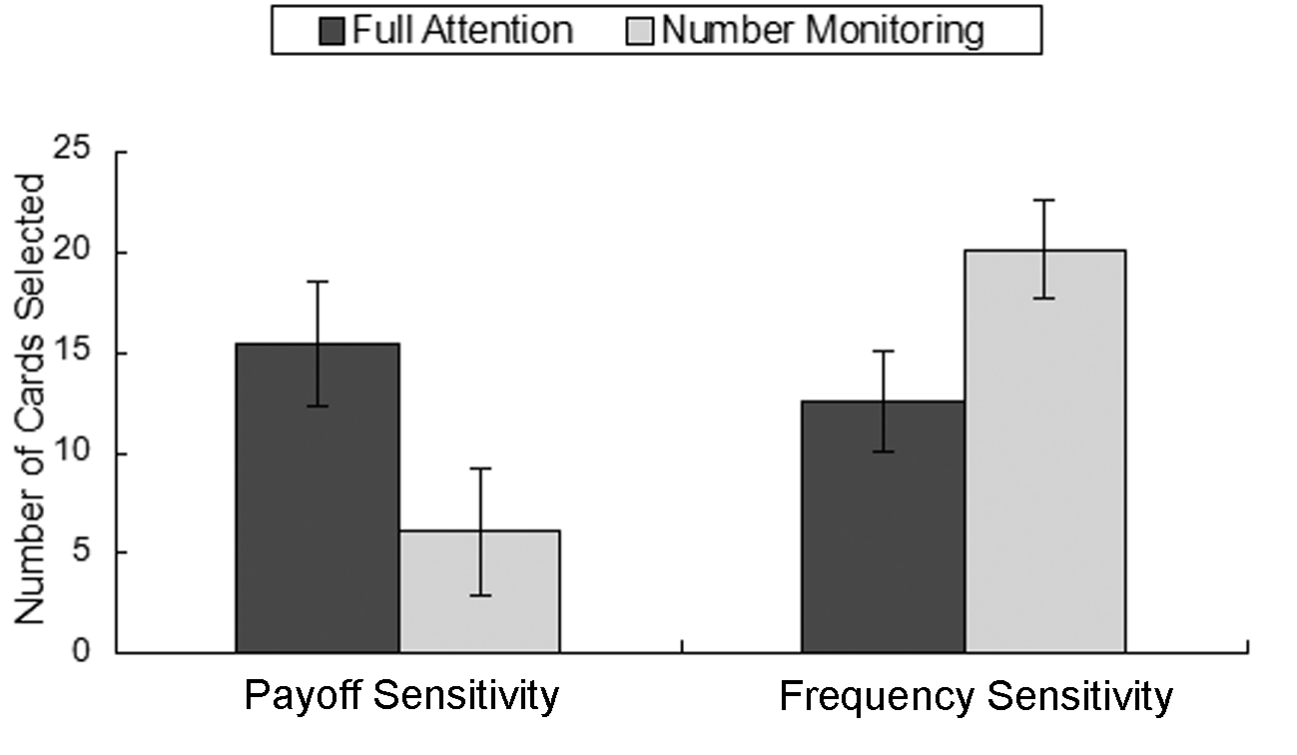
Sensitivity between groups.

Two one-way ANOVAs were conducted to identify differences between the full attention and number monitoring groups regarding both payoff and frequency sensitivity. The results showed differences in both payoff sensitivity *F(*1, 58) = 4.36, *p* = .03, *η^2^* = .07 and frequency sensitivity *F*(1, 58) = 4.63, *p* = .04, *η^2^* = .07. Compared to the number monitoring group, the full attention group was influenced more by payoff amounts. In contrast, the number monitoring group was more sensitive to loss frequency than was the full attention group (see Figure 1).

**Figure 2:**
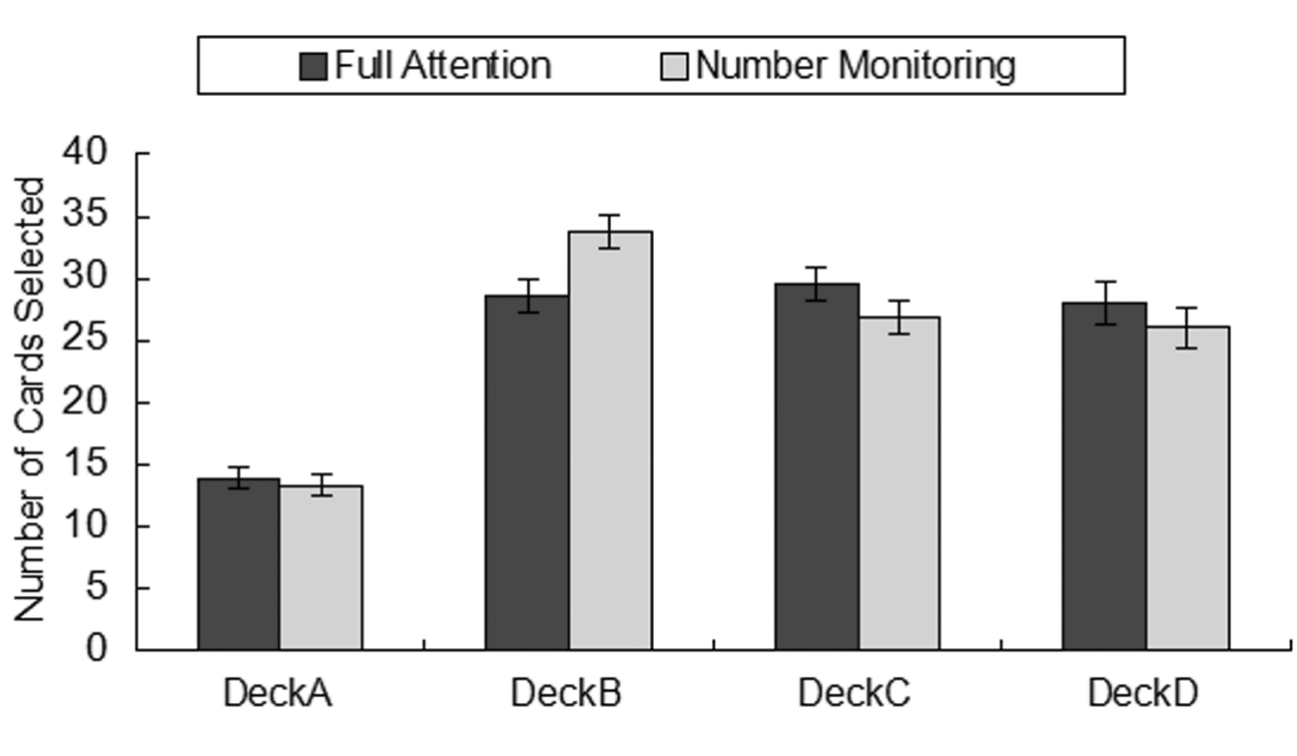
Draws by deck.

A series of one-way ANOVAs were used to analyze differences between the groups (i.e., full attention group and number monitoring group) on the different decks. A Bonferroni correction was used to correct for inflated chances of Type 1 error caused by repeated comparisons, resulting in a *p* criterion of .0125. The results indicated that there was a significant difference between groups only for Deck B, *F*(1, 58) = 7.24, *p* = .009, *η^2^* = .11, showing that disrupting attentional resources can lead to an increased preference for selecting Deck B (see Figure 2).

**Figure 3:**
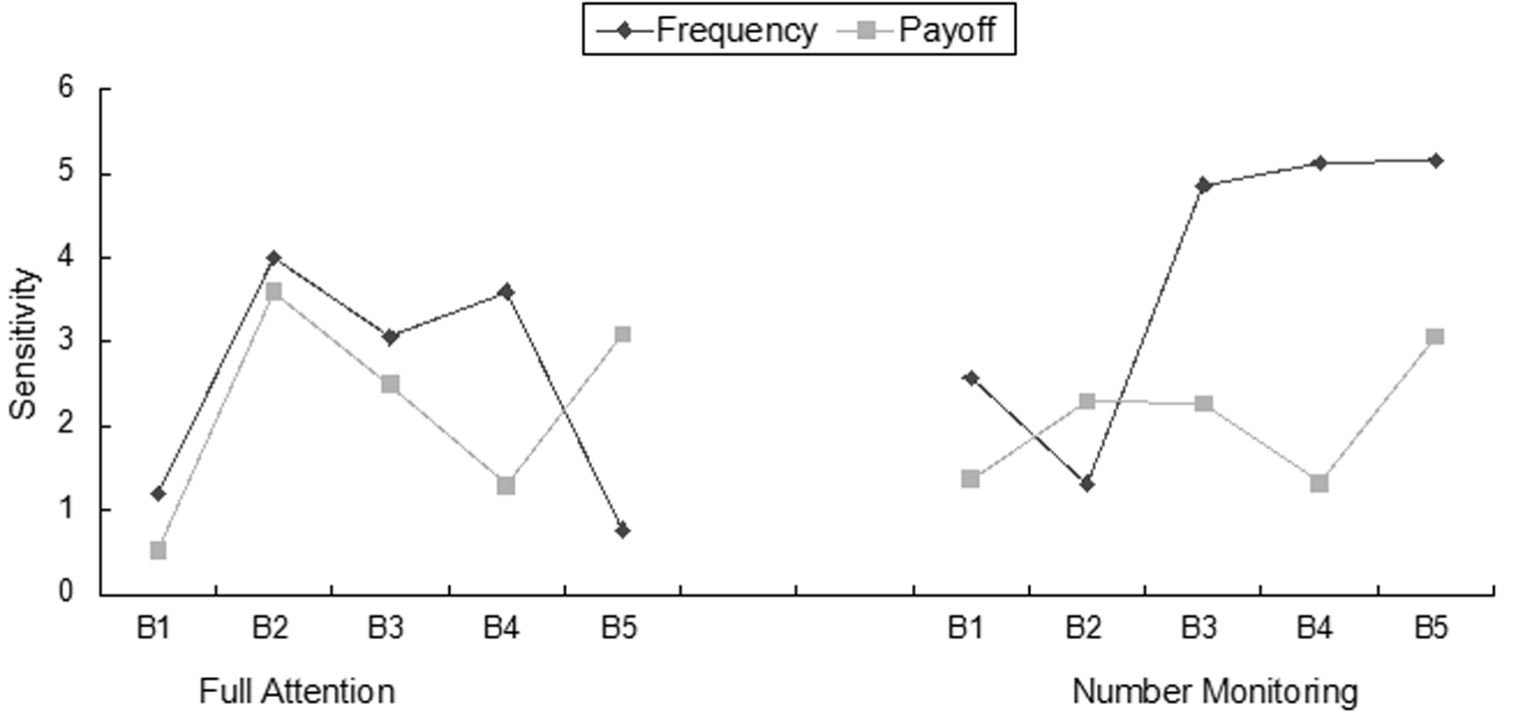
Group performance across blocks.

To further explore how cognitive load impacted frequency and payoff sensitivity, we next scored participants’ frequency sensitivity and payoff sensitivity by block (5 blocks of 20 responses). We conducted mixed ANOVAs for both payoff and frequency sensitivity. For frequency sensitivity, there was a main effect of Block, *F*(4, 58) = 6.71, *p* = .01, *η^2^* = .10, but the interaction (Block X Group) was only marginally significant (*p* = .08). Between subjects, there was also a significant effect of group membership, *F*(1, 58) = 6.63, *p* = .01, *η^2^* = .10, with the number monitoring group having greater sensitivity. For the number monitoring group, frequency awareness changed across blocks, with higher levels of frequency awareness over the last three blocks (see Figure 3). In contrast, the full attention group demonstrated an increase in frequency sensitivity from Block 1 to Block 2, followed by a relatively stable sensitivity until the last block. In Block 5, frequency sensitivity dropped sharply. Next, we conducted a mixed ANOVA for payoff sensitivity. There was a main effect of Block, *F*(4, 58) = 2.46, *p* = .04, *η^2^* = .04, and no interaction (*p* = .23). There was also no difference between groups for payoff sensitivity (*p* = .33).

A comparison across the different blocks showed that although the full attention group tended to be more aware of the payoff amount, participants were occasionally influenced by the frequency of losses (e.g., Block 4; see Figure 3). In contrast to the number monitoring group, which was consistently more sensitive to loss frequency, the full attention group experienced changes in focus, shifting between frequency and amount. This variability supports the idea that with full cognitive resources available, participants were better able to weigh previous results and make changes in their responses. Without such resources, the number monitoring group tended to rely on a single aspect of previous responses, the frequency with which a deck produced a loss.

**Figure 4:**
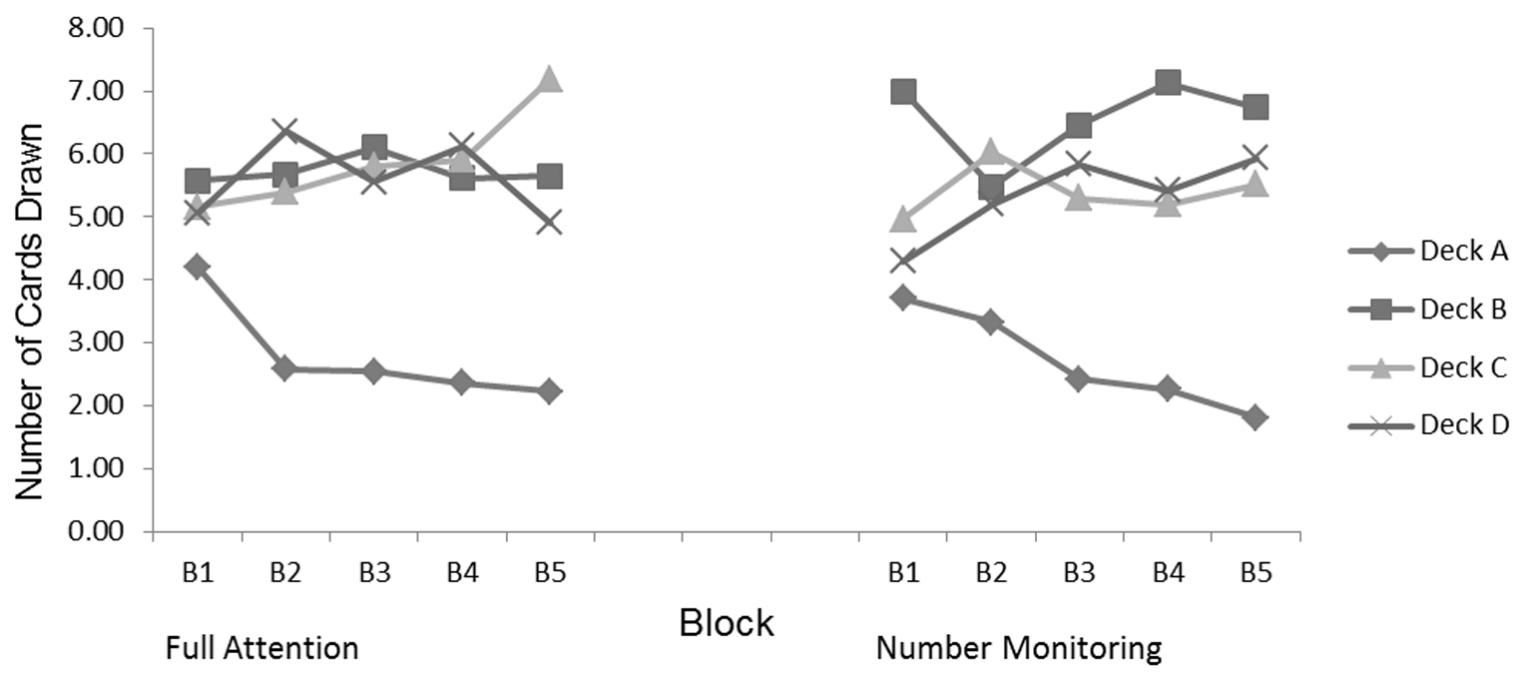
Draws per deck across blocks.

Finally, we examined deck selection by block using a series of repeated-measures ANOVAs. For the full attention group, changes across blocks in the number of cards drawn from each deck occurred only for Deck A, *F*(4, 29) = 7.55, *p* < .001, *η^2^* = . 21, and for Deck C, *F*(4, 29) = 3.59, *p* = .009, *η^2^* = .11. This group consistently selected a lower number of cards from Deck A for all but the first block. However, the difference for Deck C came in the last block of 20 draws (see Figure 4). The number monitoring group also drew fewer cards from Deck A, *F*(4, 29) = 11.15, *p* < .001, *η^2^* = . 27. However, this trend began after the second block of draws. Although both groups were able to identify Deck A as being disadvantageous and consequently avoid drawing from that deck, it took the number-monitoring group longer to make this determination. Furthermore, the full attention group drew equally from Decks B, C, and D until the final block. In that block, participants drew primarily from Deck C. This pattern indicates that the participants were able to balance payoff amount and loss frequency and adjust their draws accordingly. In contrast, the number monitoring group was not able to make this distinction, and instead relied more on frequency of losses as the guiding factor in card selection.

## Discussion

Considerable effort has been placed on understanding the processes, both cognitive and emotional, that contribute to performance on the Iowa Gambling Task. One finding that has not been fully explored is the prominent Deck B phenomenon. Research has shown that certain populations are more susceptible to this phenomenon, including children and adolescents, (Cassotti et al., 2011; Huizenga et al., 2007), older adults (Beitz et al., 2014), and certain clinical populations (Shurman et al., 2005; Toplak et al., 2005). Because these populations all have limited cognitive resources compared to older adolescents and younger adults (Cella, Dymond, Cooper, & Turnbull, 2012; Plude, Enns, & Brodeur, 1994; Salthouse, 2004; Trick & Enns, 1998), we hypothesized that limited resources might be the driving factor behind the Deck B phenomenon. Typically, research that has sought to link particular resources such as working memory, set-shifting, inhibition, and intelligence has yielded mixed results (Toplak et al., 2010) with effect sizes that have often been small (Beitz et al., 2014). Therefore, we chose to focus on attentional abilities. To test the hypothesis that impaired attention would affect deck selection, we had one group of cognitively normal participants complete the IGT under a full-attention condition, and a second group complete the task under a divided attention condition. We chose a number monitoring task for the divided attention condition because research has shown that this task has high processing demands but a low error rate (Fernandes & Moscovitch, 2000; Park et al., 1989). Therefore, the task affects initial encoding by limiting attentional resources, while minimizing the effects on other cognitive resources (e.g., working memory, inhibition).

We first compared payoff and frequency sensitivity between the full attention and number monitoring groups. Our results showed that participants in the full attention group were influenced more by payoff amounts than those in the number monitoring group, whereas participants in the number monitoring group were more sensitive to loss frequency. These findings are consistent with those of Huizenga et al. (2007) who found that two main strategies accounted for deck preference in the IGT. The more sophisticated strategy of weighing both payoff frequency and the amount of probabilistic loss required fully developed cognitive abilities and was typically seen in older adolescents and younger adults. Within groups, there was no significant difference between payoff and frequency sensitivity for the full attention group. Thus, consistent with Huizenga et al., participants with full attentional resources were able to incorporate both payoff frequency and amount into their deck selections, whereas participants with impaired attentional resources defaulted to a reliance on the frequency of losses to guide their deck choices.

Next, we examined how this focus on payoff and loss frequency affected individual deck selection. Huizenga et al. (2007) found that participants who focused on loss frequency demonstrated a preference for Decks B and D. However, Cassotti et al. (2011) found that although children and adolescents developed a bias toward selecting Deck B, there was no preference for Deck D. In the current study, participants in the number monitoring condition exhibited the prominent Deck B phenomenon. In contrast, there were no significant differences in the number of draws from Decks B, C, or D for participants in the full attention condition, findings that are consistent with those of Cassotti et al.

Our results are also consistent with those of Beitz et al. (2014) who found that both children and older adults performed poorly on the IGT, driven primarily by a tendency to focus on the frequency of losses. Similar choice patterns have been demonstrated in populations known to do poorly on the IGT, such as people with schizophrenia (Shurman et al., 2005) and those with ADHD (Toplak et al., 2005), lending support to the idea that intact cognitive resources are needed for optimal IGT performance.

Our next analysis focused on the impact of participants’ sensitivity to loss frequency and payoff amounts throughout the IGT. We divided the total number of draws into 5 blocks of 20 draws each and computed frequency and payoff sensitivity for each block. Mixed ANOVAs showed that the number-monitoring group was consistently influenced more by the frequency of the losses than by the amount of the gains. However, the full attention group tended to rely almost equally on frequency and amount until the last two blocks. In Block 4, participant choices were driven largely by loss frequency. This trend reversed in the final block with payoff amount becoming the primary concern in deck selection. These trends indicate that the two groups reacted differently to the feedback provided by the IGT. The full attention group made adjustments over time, whereas the number monitoring group tended to rely on one strategy. It may be that those in the number monitoring group did not realize the need for a strategy change, or that they realized that a strategy change was needed, but did not have, or chose not to expend, the resources needed to make the adjustment. Either way, the reduction in attentional resources influenced how participants reacted to the gains and losses.

In our final analysis, we examined each group’s deck selections across five blocks of 20 draws each. These results supported our earlier findings that the full attention group was better able to weigh both loss frequency and amount of payoffs in making deck selections. The full attention group quickly identified Deck A as being disadvantageous and tended to avoid that deck for the remainder of the task. Across Blocks 2, 3, and 4, they drew equally from Decks B, C, and D. In the final block, however, they shifted to drawing more frequently from Deck C. This shows that the full attention group was able to recognize that Deck C was advantageous and drew more cards from that deck to increase the overall gain. In contrast, it took the number monitoring group longer to recognize Deck A as being a bad deck. Once this determination was made, the number monitoring group drew equally from Decks C and D but drew much more frequently from Deck B. They apparently were influenced more by the frequency of payoffs and less so by the amount of the gain or loss. Again, these results are consistent with other findings that showed the impact of diminished cognitive resources on IGT performance (e.g., Beitz et al., 2014; Shurman et al., 2005).

Our findings also have relevance to dual systems theories of reasoning and decision making. One dual process account put forth by Stanovich and West (1999) posits that one process relies largely on rational, cognitively demanding thought, whereas the other process is fast, intuitive, and tends to be emotion based. To avoid the implication that decision-making is governed by two separate and distinct brain systems, Stanovich and Toplak (2012) later adopted the terms Type 1 and Type 2 processes. Because Type 2 processes require cognitive effort, they are affected by tasks that compete for the limited available resources, often causing a shift to using Type 1 processes. Our findings suggest that when under cognitive load, participants were unable to fully use Type 2 processes to weigh loss and gains against loss frequency, and therefore relied more on intuitive Type 1 processes. (For a review of dual process decision making see Evans, 2011)

Similarly, Reyna and Brainerd (2011) applied Fuzzy Trace theory as a dual systems account of decision making. From this perspective, gist memory is intuitive, automatic, and quick, whereas verbatim memory is more analytical and resource-consuming. Our results suggest that under full attention conditions participants were better able to use verbatim memory of previous results when making deck selections. In contrast, the number monitoring group tended to rely more on the gist memory of the gain from Deck B, when the retrieval of verbatim memory would have shown that Deck B actually yielded an overall loss.

To our knowledge, there has been no research that addresses the issue of attentional resources and decision making factors such as loss aversion. However, there is some evidence that cognitive load does impact which factors are considered by the decision maker. For example, Shiv and Fedorikhin (1999) demonstrated that under cognitive load, participants were more likely to choose an option that was highly appealing on an affective level (chocolate cake). However, in a no-load condition, the participants tended to choose the more cognitively appealing desert (fruit salad). Similarly, Biswas and Grau (2008) found that under cognitive load, consumers chose more optional features when making a purchase. The authors argued that cognitive load prevents consumers from accurately processing price-ratio information. Other research has also shown that when participants are placed under a cognitive load they tend to rely on heuristics rather than on analytical reasoning (De Neys, 2006; Gillard, Van Dooren, Schaeken, & Verschaffel, 2009; Pelham, Sumarta, & Myaskovsky, 1994). Our findings support the idea that under cognitive load, individuals tend to rely on prominent and salient features rather than engage in analytical reasoning. Therefore, our research has real-world applications, perhaps extending to areas such as consumer research and behavioral economics.

One potential limitation of the current study is that we only used one type of task (i.e., number monitoring) to induce cognitive load. Therefore, we do not know the extent to which a more demanding divided-attention task would affect sensitivity to loss frequency and selections from Deck B. Another limitation is that we did not include any physiological measures (e.g., skin conductive responses) that might reveal how divided attention affects emotional reactions to deck outcomes.

Despite these limitations, we provide a new explanation for the prominent Deck B phenomenon. Typically the preference for Deck B that was observed in certain populations was explained as an artifact of the task’s reward/punishment schedule (Lin et al., 2007). However, our results indicate that the availability of cognitive resources is necessary for participants to engage in the more complex decision strategy of weighing both the frequency of losses and the probabilistic gain or loss associated with a deck. When participants’ attentional resources were taxed by a number monitoring task, they defaulted to using loss frequency to guide their deck choices. This basic strategy, in turn, resulted in these participants drawing more often from Deck B than from any other deck. These results demonstrate, for the first time, that limited attentional resources may be sufficient to explain the preference for Deck B among certain populations.
